# Risk prediction model of physical frailty for a rural older population: a cross-sectional study in Hunan Province, China

**DOI:** 10.3389/fpubh.2025.1525580

**Published:** 2025-02-28

**Authors:** Xiuyan Guo, Chunhong Shi

**Affiliations:** 1 School of Nursing, Jiujiang University, Jiujiang, China; 2 School of Nursing, Xiangnan University, Chenzhou, China

**Keywords:** physical frailty, rural, older adults, FRAIL scale, predictive model

## Abstract

**Introduction:**

Physical frailty is a common medical syndrome characterized by low muscle strength, low endurance, and reduced physiological function that leads to significantly negative health outcomes in older adults. This study investigated the risk variables among rural older adults in Hunan Province, China, and developed a physical frailty prediction model to inform policymaking to enhance their health and well-being.

**Methods:**

This study was conducted from July 22 to September 3, 2022. A total of 291 participants were recruited using stratified cluster random sampling from five large villages in Hunan Province. Frailty screening was performed based on the Fatigue, Resistance, Ambulation, Illnesses, and Loss of Weight (FRAIL) scale, Geriatric Depression Scale 15-item version (GDS-15), Falls Efficacy Scale-International (FES-I), and Mini Nutrition Assessment-Short Form (MNA-SF). A logistic regression analysis was performed to identify the predictive factors for physical frailty and develop a physical frailty prediction model based on the area under the receiver operating characteristic (ROC) curve (AUC), sensitivity, specificity, and Youden index.

**Results:**

The physical frailty prevalence among rural older adults in Hunan Province was 21.31% (*n* = 62). Household income and expenditure [odds ratio (OR): 1.826, 95% confidence interval (CI): 1.142–2.918], physical exercise frequency (OR: 1.669, 95% CI: 1.137–2.451), depressive symptoms (OR: 9.069, 95% CI: 3.497–23.516), and fear of falling (OR: 3.135, 95% CI: 1.689–5.818) were identified as significant predictors of physical frailty in rural older individuals. The AUC for the frailty predictive model was 0.860 (95% CI: 0.805, 0.914). The sensitivity and specificity at the optimal cutoff value were 80.6 and 76.0%, respectively, with a Youden index of 0.566.

**Conclusion:**

The prediction model constructed in this study demonstrated promise as a potential tool for evaluating physical frailty risk in older adults, which can contribute to healthcare providers’ screenings for high-risk populations. Further multidimensional and experimental intervention studies should be conducted to prevent the occurrence and delay the progression of physical frailty in older adults.

## 1 Introduction

Population aging has become a global challenge. Every country is experiencing the growing proportion of older adults in the population and China is no exception (1). An older adult is defined by the United Nations as a person who is over 60 years of age (2). Based on a China Bureau of Statistics report, people aged over 60 years has reached 296 million, accounting for 21.2% of the total population (3). Older people become frail due to physiological or psychological factors such as aging and illness (4). There are a number of older people in the Chinese rural regions, in rural areas the older population accounted for 17.72% of the total population, whereas in urban areas it accounted for 11.11%. Older population in rural area is a critical attention to achieve an objective of health care equity and healthy aging (5).

Frailty phenotype was initially defined by Fried et al. (6) and refers to an age-related medical syndrome increasing individual’s excessive vulnerability for developing increased dependency and/or death. Physical frail older people are mainly manifested in the aspects of lower muscle strength, lower endurance, and decreased physiological function (7). A meta-analysis found that frailty prevalence in rural older residents was 18%, greater than the global estimate of 10.7% (8). Previous studies have compared the frailty prevalence between rural and urban regions (9–14), with a 1.5 times higher frailty prevalence (10). Physiological function frail leads to inadequate basic daily activities ability and is susceptible to chronic diseases or comorbidity (15). These health conditions induce a higher risk of negative health outcomes such as hospitalization or nursing home admission, premature death (16), and Medicare demand with increased costs for individuals and health systems (annual cost $3,781 healthy vs. $10,755 frail) (17). Many countries consider addressing physical frailty as a priority to reduce the medical burden (18, 19).

Early screening of high-risk populations is important for early intervention to delay the onset and progression of physical frailty (20). The risk levels of physical frailty older people were affected by a range of factors and conditions such as age (21, 22), educational level (23), depression, fear of falling, nutritional status (24), and loneliness (25). Previous studies have attempted to develop frailty prediction models for developing countries, particularly for use in rural areas. However, their use is context dependent. During the urbanization process in China, population migration has produced sociodemographic profiles for the rural older population that are influenced by various factors such as economic development (26), distribution of health resources, and individual lifestyles. Little attention has been paid to rural residents and risk identification models of physical frailty have not been applied in China. A predictive model enables the accurate calculation of risk and identification of high-risk rural residents. This study aims to identify risk variables and create a predictive model of physical frailty for rural older residents.

## 2 Materials and methods

### 2.1 Study design and participants

This cross-sectional observational study was conducted to develop a risk predictive model of physical frailty among rural populations in Hunan Province, central China. We report the results of this study in accordance with the Strengthening the Reporting of Observational Studies in Epidemiology (STROBE) checklist guidelines (Supplementary Appendix 1).

Stratified cluster random sampling was employed to recruit the participants. Specially, five large villages were randomly selected in this study from Yueyang City, Yongzhou City, Zhuzhou City, Chenzhou, City and Huaihua City. Participant inclusion criteria were: ① resided in the village at least 6 months; ② Age 60 years or older; ③ capable of communication; and ④ voluntarily agreed to participate in the study. Older adults with severe diseases, mental disorders, or serious audio-visual impairments were excluded so as to the accuracy of the study findings.

According to Kendall’s sample size calculation method (27), the sample size should be 5–10 times the number of independent variables. Using three scales and 17 items, and assuming a 20% sample loss, the required sample size for this study was 120–240. Ultimately, 291 valid questionnaires were obtained to meet the sample size requirements.

### 2.2 Survey tools

The questionnaire employed in this study consisted of five parts: a general information questionnaire, the Fatigue, Resistance, Ambulation, Illnesses, & Loss of Weight (FRAIL) scale, Geriatric Depression Scale 15-item version (GDS-15), Falls Efficacy Scale-International (FES-I), and Mini Nutrition Assessment-Short Form (MNA-SF).

#### 2.2.1 General information questionnaire

This section included 11 demographic items (age, sex, ethnicity, occupation, body mass index, education level, marital status, smoking status, alcohol consumption, family income, and living conditions) and 6 related factors (social activities, physical exercise, intellectual activities, falls in the past year, chronic diseases, and types of drugs administered).

#### 2.2.2 FRAIL scale

The FRAIL scale was developed by the International Academy of Nutrition, Health, and Aging (28). It consists of five items: fatigue, resistance, ambulation, illnesses, and loss of weight. Each item is scored as 0 or 1, with the total score ranging from 0 to 5. According to the total scores are categorized as not-frailty (≤2 points), and frailty (≥ 3 points). In this study, the Cronbach’s *α* coefficient of this scale was 0.605.

#### 2.2.3 GDS-15

The GDS-15 was developed by Sheik and Yesavage (29) in the 1980s, and translated into Chinese by Tang et al. (30). The GDS-15 has 15 items, including 10 affirmative (“yes” is 1 point and “no” is “no”) and 5 negative items (“yes” is 0 points and “no” is 1 point). The total GDS-15 score is 0–15 points, the cutoff point of ≥8 indicates the presence of depressive symptoms, and a higher score indicates more severe depressive symptoms. In this study, the Cronbach’s *α* coefficient of this scale was 0.753.

#### 2.2.4 FES-I

The FES-I was revised by Yardley et al. (31, 32) and translated into Chinese by the researcher Guo et al. (33). It consists two dimensions: indoor physical activities (10 items) and outdoor physical activities (6 items). Items are scored using a Likert 4-point scale, ranging from “not at all worried” to “very worried,” with scores from 1 to 4. The total score ranges from 16–64; higher scores indicate a higher degree of fear of falling. Specifically, scores of 16–31 indicate low, 32–47 indicate moderate, and 48–64 indicate high fear of falling. In this study, the Cronbach’s *α* coefficient of this scale was 0.870.

#### 2.2.5 MNA-SF

The MNA-SF was developed by Kaiser et al. (34) and then translated into the Chinese version by He et al. (35). The MNA-SF comprises six indicators: body mass index, decline in food, intake, stress, mobility, weight loss in the prior 3 months, and neuropsychiatric symptoms; a total score ranges from 0–14 points and scores are categorized as normal nutritional status (12–14 points), risk of malnutrition (8–11 points), and malnutrition (<8 points). The MNA-SF is an effective nutritional screening tool suitable for older adults because it demonstrates good sensitivity and specificity in identifying malnutrition among this population (36, 37). In this study, the Cronbach’s *α* coefficient of this scale was 0.619.

### 2.3 Data collection

The questionnaires were collected face-to-face from July 22 to September 3, 2022. Before data collection, we recruited 12 sophomore nursing students as investigators who were divided into six groups (2 investigators in each group). The primary researcher taught them the nursing research curriculum and provided instructions for the questionnaire survey guidelines, quantitative research design, and data collection methods. The primary researcher printed written questionnaires and brought all groups of investigators to the five villages separately.

When collecting data, investigators used unified guidelines to ask participants about all items and filled in the questionnaires with their answers. If the older adults did not understand the meaning of the items or express their opinions accurately during the survey, their families or caregivers assisted in explaining them until they understood the meaning. After completing the questionnaire, each participant received a small gift (one toothbrush, one tube of toothpaste, and one bag of washing powder) to express gratitude. In this study, 306 questionnaires were collected; 15 invalid questionnaires that showed incomplete or incorrect information were excluded. Thus, 291 valid questionnaires were obtained with an effective rate of 95.10%.

### 2.4 Ethical considerations

This study was approved by the Institutional Review Board of the Medical Ethics Committee of Xiangnan University (registration number: 2022010). Before collecting data, all participants were informed about the purpose, process, and potential risks of the study and then were asked to provide written informed consent. They were allowed to withdraw from the study at any time without explanation. Their information was kept confidential and used only for research purposes. This study conformed to the guidelines of the 1995 Declaration of Helsinki (and its revised editions since 2000).

### 2.5 Data analysis

SPSS version 25.0 (IBM Corp., Armonk, NY, USA) was used to analyze data for all statistical analyses. A *p*-value <0.05 determined significance. The continuous data were statistically described by means and standard deviations, while the categorical data by frequency and percentage. For the univariate analysis, the *χ*2 test was employed. A binary multivariable logistic regression model was performed to estimate physical frailty risk and considered predictive factors as the significant factors obtained in the bivariate analysis. The discriminatory power of the risk model was evaluated based on the area under the receiver operating characteristic (ROC) curve (AUC), sensitivity, specificity, and Youden index. The Hosmer–Lemeshow test was used to analyze the model fit.

## 3 Results

### 3.1 Demographic characteristics and clinical characteristics

**Figure 1:**
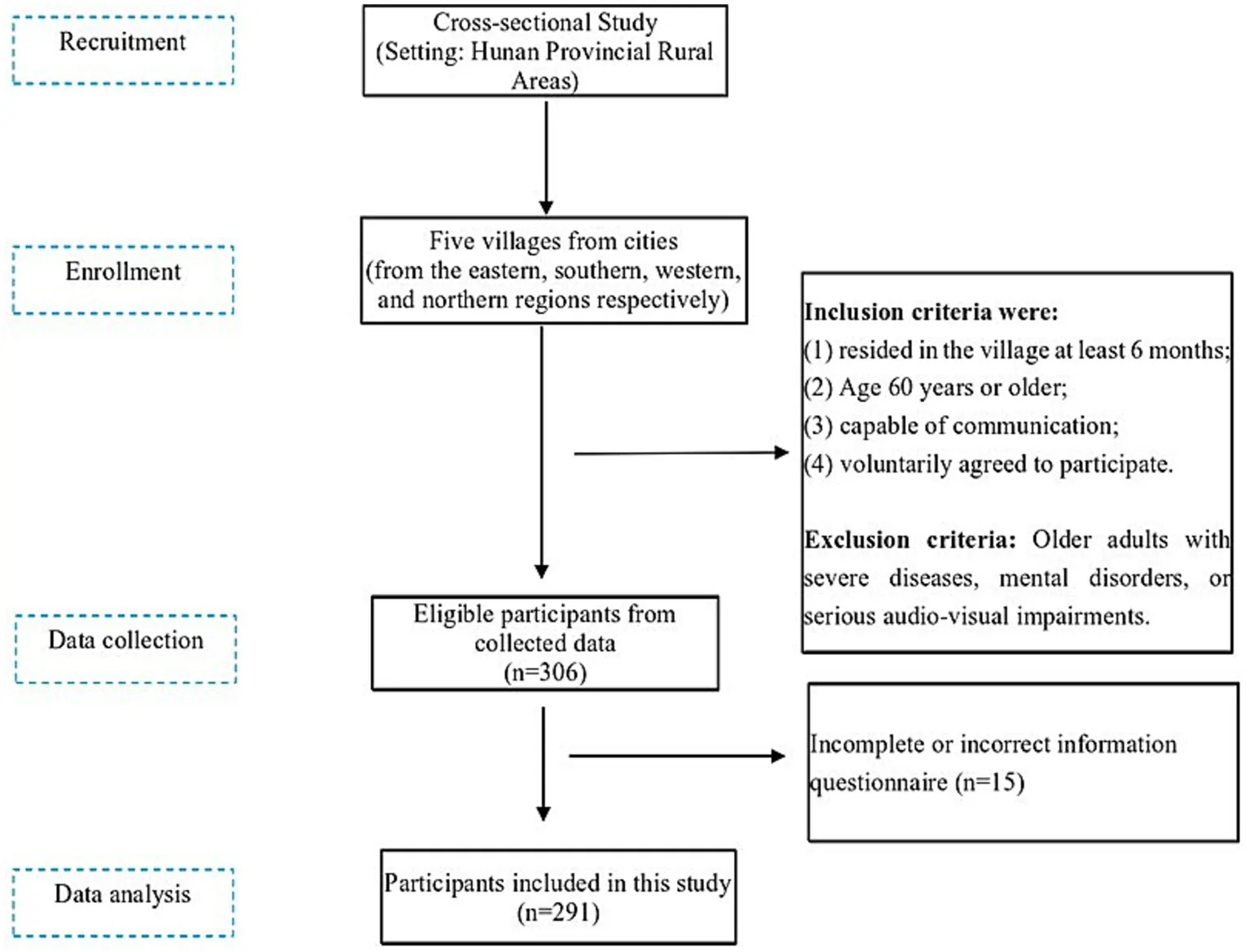
Flow diagram of recruitment procedure.

A total of 291 older people were investigated in this study. Figure 1 illustrates the recruitment process of participating rural older adults. Among them, there were 123 males (42.27%) and 168 females (57.73%); the age ranged from 60 to 94 years, with an average of (70.42 ± 7.09) years. 102 older adults (35.05%) had never attended school, 142 (48.80%) with primary school, and 47 (16.15%) with junior high school or above. In terms of family income, 143 older adults (49.14%) had income exceeding expenses, 96 (32.99%) with a balanced income and expenses, and 52 (17.87%) with expenses exceeding income. The prevalence of depressive symptoms was 12.37% (*n* = 36), and that of older adults at risk of malnutrition was 224 (76.97%). The fear of falling score was (29.69 ± 11.11), with an average score of (1.86 ± 0.69). The participant characteristics are listed in Table 1.

**Table 1:** Socio-demographic characteristics and physical frailty prevalence among the rural older population in Hunan Province.

Variables		Frailty (%) (*n* = 62)	Non-frailty (%) (*n* = 229)	*χ* ^2^	*p*-value
Age in years	60–69	24 (16.67)	120 (83.33)	21.485	< 0.001*
	70–79	19 (17.43)	90 (82.57)		
	≥80	19 (50.00)	19 (50.00)		
Sex	Male	27 (21.95)	96 (78.05)	0.053	0.818
	Female	35 (20.83)	133 (79.17)		
Ethnicity	Han	59 (20.92)	223 (79.08)	0.720	0.396
	Minority	3 (33.33)	6 (66.67)		
Occupation	Farmer	55 (20.92)	192 (79.08)	0.900	0.343
	Non-farmer	7 (15.91)	37 (84.09)		
BMI (kg/m^2^)	Low weight	11 (27.50)	29 (66.67)	1.938	0.379
	Normal	36 (18.95)	154 (79.08)		
	Overweight	15 (24.59)	46 (84.09)		
Educational level	No school	22 (21.57)	80 (78.43)	0.194	0.908
	Elementary school	29 (20.42)	113 (79.58)		
	Junior school or above	11 (23.40)	36 (76.60)		
Marital status	Married/Reconciled	35 (17.33)	167 (82.67)	6.237	0.013
	Unmarried/Divorced/Widowed /Separated	27 (30.34)	62 (69.66)		
Smoking status	Never	41 (18.98)	175 (81.02)	6.677	0.035
	Current	15 (23.44)	49 (76.56)		
	Quit	6 (54.55)	5 (45.45)		
Alcohol consumption	Never	45 (22.06)	159 (77.94)	6.532	0.038
	Current	10 (14.08)	61 (85.92)		
	Quit	7 (43.75)	9 (56.25)		
Family income	Income more than expenditure	17 (11.89)	126 (88.11)	33.232	< 0.001
	Balance	19 (19.79)	77 (80.21)		
	Expenditure more than income	26 (50.00)	26 (50.00)		
Living conditions	Living with spouse	15 (14.85)	86 (85.15)	8.725	0.033
	Living with children	25 (32.05)	53 (67.95)		
	Living with spouse and children	16 (17.78)	74 (82.22)		
	Living alone	6 (27.27)	16 (72.73)		
Frequency of participating in social activities	≥ 1 time/week	17 (63.73)	59 (36.27)	0.069	0.792
	<1 time/week	45 (69.32)	170 (30.68)		
Frequency of participating in physical exercise	Everyday almost	1 (1.32)	75 (98.68)	37.015	< 0.001^*^
	3–5 times/week	11 (18.03)	50 (81.97)		
	1–8 times/week	15 (22.06)	53 (77.94)		
	<1 time/month	35 (40.70)	51 (59.30)		
Participation in intellectual activities	Yes	20 (15.04)	113 (84.96)	5.740	0.017
	No	42 (26.58)	116 (73.42)		
Falling occurred within 1 year	Yes	13 (34.21)	25 (65.79)	4.341	0.037
	No	49 (19.37)	204 (80.63)	1.099	0.295
Numbers of comorbidity of chronic disease	No	8 (8.42)	87 (91.58)	20.296	< 0.001^*^
	1	29 (22.31)	101 (77.69)		
	≥ 2	25 (37.88)	41 (62.12)		
Numbers and types of drugs administered	None	21 (12.96)	141 (87.04)	16.645	< 0.001^*^
	1	26 (35.62)	47 (64.38)		
	≥ 2	15 (26.79)	41 (73.21)		
Depression	Yes	26 (72.22)	10 (27.78)	63.523	< 0.001^*^
	No	36 (14.12)	219 (85.88)		
Nutritional status	Normal	6 (13.95)	37 (86.05)	10.196	0.006
	At risk of malnutrition	45 (20.09)	179 (79.91)		
	Malnutrition	11 (45.83)	13 (54.17)		
FOF level	Low	11 (7.38)	138 (92.62)	61.619	< 0.001^*^
	Middle	33 (27.97)	85 (72.03)		
	High	18 (75.00)	6 (25.00)		

BMI, body mass index; FOF, fear of falling.

^*^p < 0.001.

### 3.2 Prevalence of physical frailty and risk factors

The prevalence of physical frailty was 21.31% (*n* = 62) among the 291 rural older adults in Hunan Province. There were significant differences in the frailty of rural older people with age, marital status, smoking status, alcohol consumption, family income and expenditure, living conditions, frequency of participating in physical exercise, participating in intellectual activities, falling frequency within one year, number of comorbidities for chronic diseases, number of types of drugs administered, depressive symptoms, nutritional status, and fear of falling (all *p* < 0.001).

### 3.3 Predictive factors associated with physical frailty

Before the stepwise binary logistic regression, all independent variables were assigned points, as presented in Table 2. The results showed that family income, physical activity, depressive symptoms and the fear of falling were the main predictive factors affecting of older people (*p* < 0.001) (Table 3).

**Table 2:** Assigned points of variables in the logistic regression analysis model.

Variables	Assigned points
Age (years)	60–69 = 1; 70–79 = 2; ≥ 80 = 3
Marital status	Married/reconciled = 1; Unmarried/divorced/widowed/separated = 2
Smoking status	Never = 1; Current = 2; Quit = 3
Alcohol consumption	Never = 1; Current = 2; Quit = 3
Family income	Income more than expenditure = 1; balance = 2; expenditure more than income = 3
Living conditions	Living alone = 1; Living with spouse = 2; Living with children = 3; Living with spouse and children = 4
Frequency of participating in physical activities	Almost every day = 1; 3–5 times/week = 2; 5–8 times/month = 3; <1 time/month = 4
Participation in intellectual activities	No = 1; Yes = 2
Fall occurred within prior year	No = 1; Yes = 2
Number of chronic diseases	No = 1; 1 Type = 2; ≥ 2 Type = 3
Types of drugs administered	No = 1; 1 Type = 2; ≥ 2 Type = 3
Depression symptoms	No = 1; Yes = 2
Nutritional status	Normal = 1; Risk of malnutrition = 2; Malnutrition = 3
Fear of falling	Low = 1; Middle = 2; High = 3

**Table 3:** Predictive risk factors associated with physical frailty for the rural older adults by the stepwise binary logistic regression.

Variables	*β* value	SE	Wald *χ*^2^	OR (95% CI)	*p*-value
Constant	−8.477	1.000	71.829	-	< 0.001
Family income	0.602	0.239	6.332	1.826(1.142–2.918)	0.012
Frequency of participating in physical activities	0.512	0.196	6.833	1.669(1.137–2.451)	0.009
Depression symptoms	2.205	0.486	20.568	9.069(3.497–23.516)	< 0.001
Fear of falling level	1.143	0.316	13.113	3.135(1.689–5.818)	< 0.001

CI, confidence interval; OR, odds ratio; SE, standard error.

### 3.4 Effectiveness of risk prediction model of physical frailty

**Figure 2:**
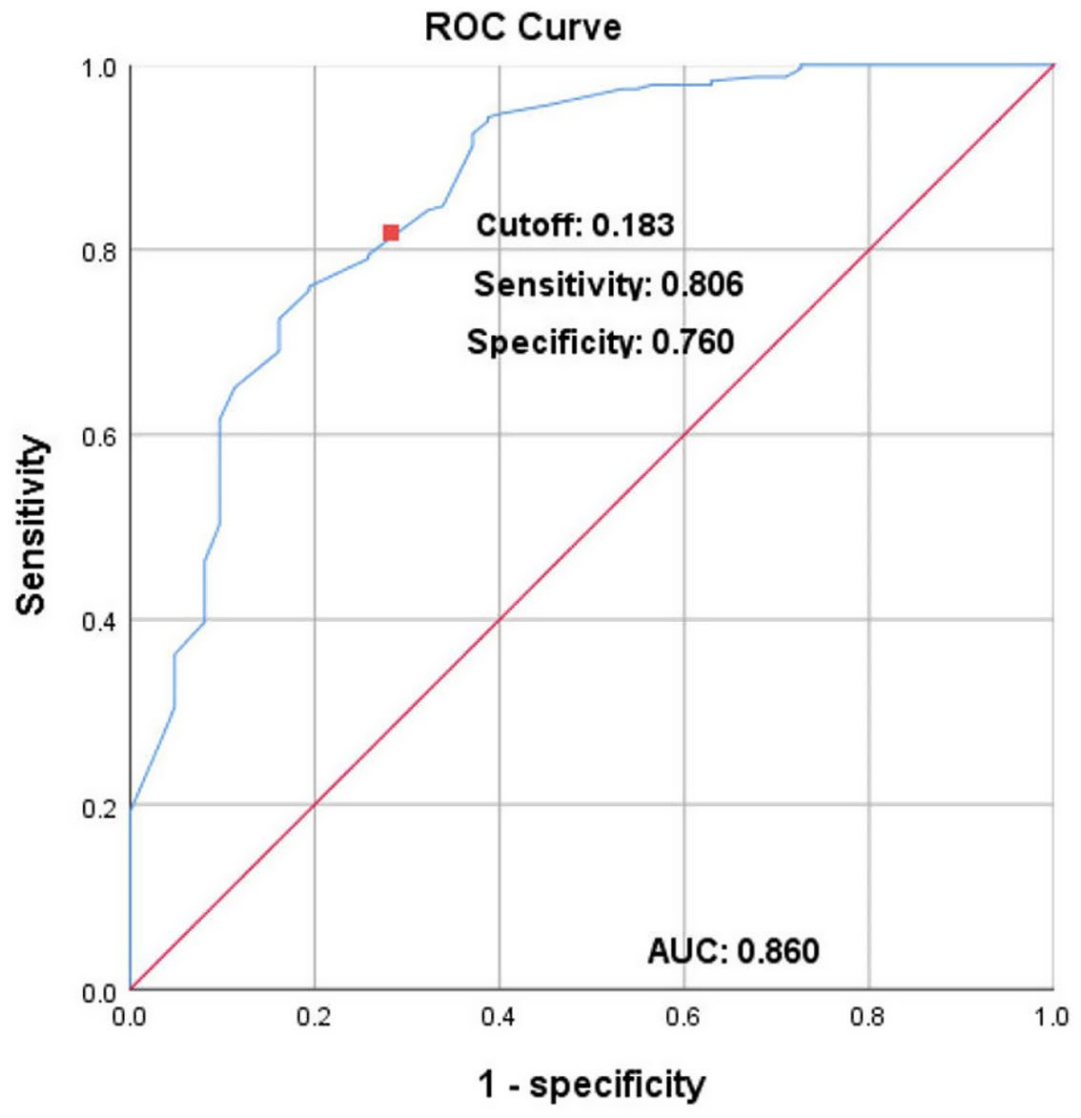
ROC curve for the physical frailty risk prediction model among older rural residents. The horizontal coordinate is 1-specificity, and the vertical coordinate is sensitivity. AUC, area under the curve; ROC, receiver operating characteristic.

Based on the significant variables and assigned values, the final risk predictive model of physical frailty was Y = −8.477 + 0.602 family income + 0.512 physical activity + 2.205 depression + 1.143 fear of falling. The area under the ROC curve was 0.860 (95% CI: 0.805–0.914). The optimal cutoff value was 0.183, corresponding to the maximum Youden index of 0.566. At this cutoff, the sensitivity and specificity values were 80.6% and 76.0%, respectively (Figure 2). The Hosmer–Lemeshow test did not indicate evidence of lack of fit (*χ*^2^ = 8.461, *p* = 0.294).

## 4 Discussion

This study established a risk prediction model for physical frailty among rural older residents, which is an essential tool in this era of an aging society. As the profile of rural older residents’ changes, it is of utmost importance to provide a scientific model to identify residents in the early frailty stage.

The results indicated that frailty prevalence among the rural older people aged 60 years and older was 21.31%. The prevalence of rural frailty varies among countries. The prevalence rate in our study is similar to those in Vietnam (38) and Japan (39), higher than that of Malaysia (40) and Tanzania (41), and lower than those of Brazil (42) and the Republic of Korea (43). In addition, among the Chinese older people, physical frailty prevalence was much higher for those dwelling in rural areas of Hunan Province than those in Beijing (44) and Shanghai (10). The high prevalence of physical frailty in rural areas is prone to a wide range of adverse outcomes. Paying attention to frail older people was one of important measures to minimize adverse events (45). This finding calls for action to identify populations at high risk in order to prevent physical frailty at an early stage.

Several factors are associated with the prevalence and progression of physical frailty. Predictive factors are context dependent and vary. This study identified low income, physical activity, depression, and fear of falling as predictive factors for physical frailty among rural older residents.

Low income in rural regions, characterized by lower socioeconomic status and education levels. This is consistent with the findings of Ahmad et al. (40) and Huang et al. (14). The results of this study revealed that majority of rural older people had received a lower level of education, with 83.85% having no formal schooling or only completed elementary school. This percentage was higher than educational level of nationwide older people, as reported in the 2021 Chinese General Social Survey (CGSS) (46, 47). Older adults with lower education exhibited higher rates of physical frailty compared to those with higher educational levels (48). Wongtrakulruang et al. (49) found that a lower education level, specifically primary school education or less, was associated with an increased risk of physical frailty. Low educational level in rural area may lead to lower family income for older people. Family income is the basic guarantee for better living. Older people with a high family income not only enjoy superior and comfortable living conditions but also have high quality nutritional intake, and high utilization rate of health services, which enables timely and effective treatment of diseases; 89% of rural older people suffer from one type of chronic disease or more (50). Worldwide, the prevalence rates appear to be higher in less developed environments and lower in more developed countries (51).

The findings of this study showed that depressive symptoms were another predictive factor of physical frailty in rural older individuals, which is consistent with the results of a study by Soysal et al. (52). Depression is a common cognitive factor. When depressive symptoms persist at high levels, they subsequently lead to cognitive decline as well as to the development or worsening of the physical frailty syndrome in older adults (53). The depression and physical frailty, in conjunction with environmental factors (e.g., low education, unhealthy dietary patterns, low physical activity) may negative influence on cognitive frailty (54), even brain aging. Ruan et al. (55) proposed that physical frailty contributed to cognitive impairment of cognitive frailty. Their interaction primarily rises from the fact that physical frailty and cognitive impairment share similar pathophysiological mechanism, which produces chronic inflammatory, oxidative stress, and mitochondrial dysfunction (52–55). Inflammatory cytokines play a crucial role in mediating cognitive frailty, meanwhile, high concentrations of inflammatory penetrate the blood–brain barrier and impact skeletal muscles, causing a reduction in muscle mass and strength, along with impaired functionality (56). This sequence of events ultimately gives rise to the onset of physical frailty. Additionally, individuals with depressive symptoms usually engage in less physical activity, have poor dietary intake (57), and are reluctant to participate socially, which increase the probability of physical frailty. In this study, 85% of older rural adults were at risk of or had malnutrition and social participation (74%) less than 1 time per week. Gaspar et al. (58) highlighted on the dimension of laser activities and free time as protective factors against cognitive impairment. It’s necessary for the rural health government to provide a social platform to promote social and cultural activities for rural residents, which enriches their spiritual life to improve depressive symptoms.

Physical activity was correlated with physical frailty progression; the prevalence of physical frailty was lower in the moderate-intensity exercise group than in the sedentary or low-intensity exercise group, which is supported by the longitudinal cohort study of Rogers et al. (59). Regarding interventions based on physical activity, different kinds of exercises has been found to improve physical function and muscle strength, such as resistance exercise, Chinese traditional mind body exercise, exergaming-based exercise and Otago exercise program (60–62). It is possible that older rural people maintain their livelihoods through some form of labor, indirectly strengthening their physical health to reduce the occurrence of physical frailty. Accordingly, physical activity, the most effective non-pharmacological intervention, enhances cognition, physical function, and mental health in older persons with frailty, providing evidence to promote future exercise programs to prevent the onset and progression of physical frailty in older adults (18). It is suggested to encourage older people to actively participate in labor or physical activity when possible.

With their fear of falling, the likelihood of developing physical frailty in rural older adults increases (63, 64). This fear of falling may have an inhibitory effect on self-efficacy (65), making older people who were previously active less energetic. Increased risk of falling may lead to reduced physical activity, but also to reduced engagement in social activities and increased social isolation, which could lead to further cognitive and functional impairment (48). Therefore, it is important to psychologically counsel older people with frailty about their fear of falling and implement interventions to prevent falls and improve their health status.

The risk-predictive model in the present study indicated good discrimination and could identify high-risk physical frailty for rural older people. This identification provides scientific evidence for appropriate interventions of rural older adults with high frailty risk to avoid adverse outcomes.

The strength of this study lies in its focus on predictive factors for frail older individuals and good efficiency of the physical frailty predictive model in rural areas to expand our understanding of frailty in different specific rural regions given the uneven distribution of the population. However, several limitations should be acknowledged. Firstly, despite the methodologically and scientifically justified the sample size, the fact of participants chosen from each village by cluster sampling within five municipalities of Hunan Province may limit the generalizability of the results to a broader rural population. Future studies should aim to expand the sample size and geographically diversify the participants to enhance external validity of the findings. Ideally, such studies could adopt a longitudinal research design to provide more robust evidence. Secondly, this study used a cross-sectional design, making it difficult to infer causality. Longitudinal studies are needed to track participants over time and provide more empirical evidence to support targeted interventions aimed at preventing or delaying the onset of physical frailty in rural older people. Thirdly, while this study has identified several important predictive factors for frailty, a wider range of potential factors should be incorporated to identify frailty to obtain more representative results in the future research. This would provide valuable evidence for physical frailty prevention programs in rural areas. Finally, the slightly insufficient Cronbach’s alpha values were obtained for the Chinese-adapted MNA-SF instrument and the FRAIL scale (0.605), indicating potential room for improvement in the reliability of these measurement tools when applied to the Chinese population. Further research could be considered to explore methods for enhancing the reliability of these instruments in Chinese-contexts. Fourthly, the slightly insufficient Cronbach’s alpha values were obtained for the Chinese-adapted MNA-SF instrument and the FRAIL scale (0.605), indicating potential room for improvement in the reliability of these measurement tools when applied to the Chinese population. Further research could be considered to explore methods for enhancing the reliability of these instruments in Chinese-contexts. Finally, the prediction model was developed and evaluated using the same study sample, without validation in an independent external sample. Consequently, the reported discrimination and classification performance may be optimistic, and the generalizability of the model to rural older adults in other regions or settings remains uncertain. Future studies should externally validate the model in independent, preferably multicenter populations and further assess its calibration and clinical utility before routine application.

## 5 Conclusion

The study established a risk-predictive model that integrates factors such as low income, depressive symptoms, lack of physical activity, and fear of falling to identify physical frail older individuals in rural areas. Frailty is a multifaceted issue involving various factors. Collaboration among public health professionals, policymakers, and stakeholders is crucial for preventing frailty. Our model could provide a potential tool to identify rural older residents at risk of physical frailty and inform prevention programs.

## Data Availability

The raw data supporting the conclusions of this article will be made available by the authors, without undue reservation.
