# Correction: Risk prediction model of physical frailty for a rural older population: a cross-sectional study in Hunan Province, China

**DOI:** 10.3389/fpubh.2026.2010162

**Published:** 2026-10-16

**Authors:** Xiuyan Guo, Chunhong Shi

**Affiliations:** 1 School of Nursing, Jiujiang University, Jiujiang, China; 2 School of Nursing, Xiangnan University, Chenzhou, China

**Keywords:** physical frailty, rural, older adults, FRAIL scale, predictive model

There was a mistake in Figure 2 as published. An incorrect Figure 2 was provided, as data from a different model were inadvertently included. Consequently, the data presented in Figure 2 did not correspond to those reported in the main text. The corrected Figure 2 appears below.

**Figure 2:**
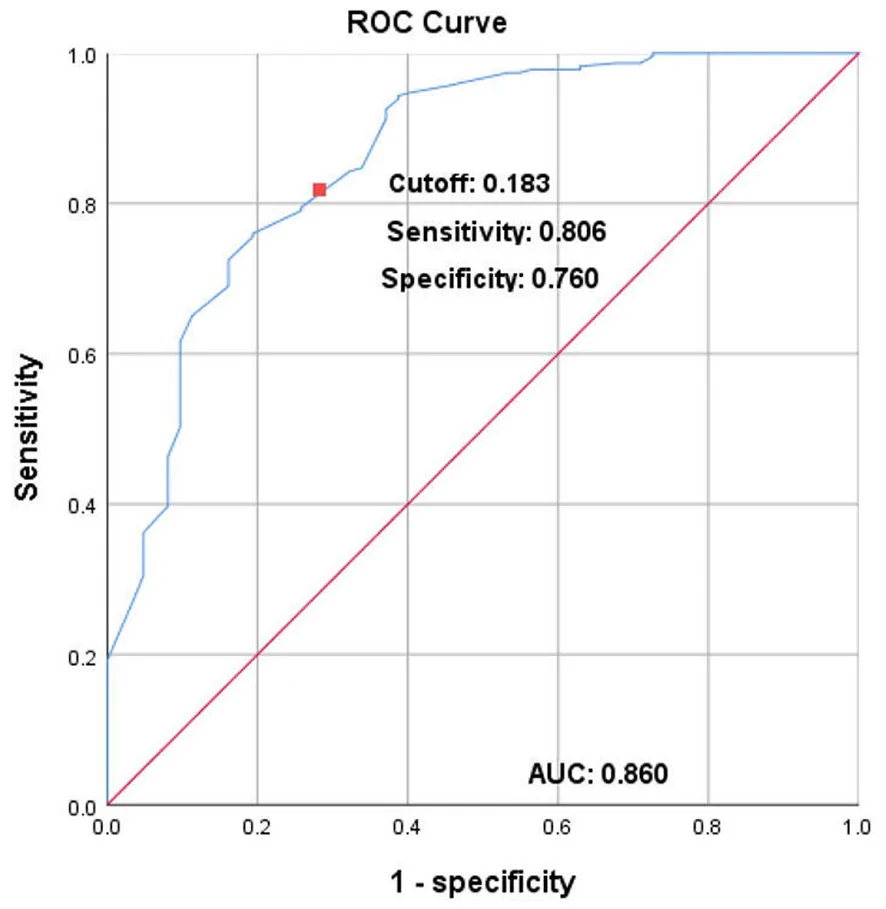
ROC curve for the physical frailty risk prediction model among older rural residents. The horizontal coordinate is 1-specificity, and the vertical coordinate is sensitivity. AUC, area under the curve; ROC, receiver operating characteristic.

White et al. was erroneously included as reference [38] and cited in **Section 3**, *Section 3.4*, Paragraph 1 and it will be removed:

“White N, Parsons R, Collins G, Barnett A. Evidence of questionable research practices in clinical prediction models. *BMC Med*. (2023) 21:339. doi: 10.1186/s12916-023-03048-6”.

In the published article, the equation and ROC statistics reported in **Section 3**, *Section 3.4*, Paragraph 1 were incorrectly provided because the five-category coding of the physical activity variable was used. The original text read:

“Based on the significant variables and assigned values, the final risk predictive model of physical frailty was *Y* = −9.076 + 0.623 family income +0.315 physical exercise +2.181 depression and + 1.165 fear of falling. The ROC curve was used to evaluate the effectiveness of the risk prediction model in the rural older population with physical frailty (Figure 2). The area under the ROC curve was 0.860 (95% CI: 0.805–0.914), which was greater than the theoretical acceptability of 0.700 (38), indicating satisfactory discriminatory performance for the risk model in identifying frail older adults in a rural setting. The optimal cutoff value of 0.201 was determined using the maximum Youden index value (0.566). The sensitivity and specificity of the optimal cutoff values were 80.6 and 76.0%, respectively. The Hosmer–Lemeshow test was used to assess goodness of fit of the predictive model; χ^2^ = 7.5668, p = 0.363 (>0.05) showed the model fitting degree is deal.”

A correction has been made to **Section 3**, *Section 3.4*, Paragraph 1, which now reads:

“Based on the significant variables and assigned values, the final risk predictive model of physical frailty was *Y* = −8.477 + 0.602 family income + 0.512 physical activity + 2.205 depression + 1.143 fear of falling. The area under the ROC curve was 0.860 (95% CI: 0.805–0.914). The optimal cutoff value was 0.183, corresponding to the maximum Youden index of 0.566. At this cutoff, the sensitivity and specificity values were 80.6% and 76.0%, respectively (Figure 2). The Hosmer–Lemeshow test did not indicate evidence of lack of fit (χ^2^ = 8.461, *p* = 0.294).”

In the published article, the study limitations did not acknowledge that the model was developed and evaluated within the same sample without external validation.

A correction has been made to **Section 4**, Paragraph 9. The following text has been added at the end of the paragraph:

“Fourthly, the slightly insufficient Cronbach's alpha values were obtained for the Chinese-adapted MNA-SF instrument and the FRAIL scale (0.605), indicating potential room for improvement in the reliability of these measurement tools when applied to the Chinese population. Further research could be considered to explore methods for enhancing the reliability of these instruments in Chinese-contexts. Finally, the prediction model was developed and evaluated using the same study sample, without validation in an independent external sample. Consequently, the reported discrimination and classification performance may be optimistic, and the generalizability of the model to rural older adults in other regions or settings remains uncertain. Future studies should externally validate the model in independent, preferably multicenter populations and further assess its calibration and clinical utility before routine application.”

The original version of this article has been updated.

